# Prolyl endopeptidase contributes to early neutrophilic inflammation in acute myocardial transplant rejection

**DOI:** 10.1172/jci.insight.139687

**Published:** 2021-03-22

**Authors:** Gregory A. Payne, Nirmal S. Sharma, Charitharth V. Lal, Chunyan Song, Lingling Guo, Camilla Margaroli, Liliana Viera, Siva Kumar, Jindong Li, Dongqi Xing, Melanie Bosley, Xin Xu, J. Michael Wells, James F. George, Jose Tallaj, Massoud Leesar, J. Edwin Blalock, Amit Gaggar

**Affiliations:** 1 Division of Cardiovascular Disease, Department of Medicine,; 2 Vascular Biology and Hypertension Program,; 3 Comprehensive Cardiovascular Center, and; 4 Program in Protease and Matrix Biology, University of Alabama at Birmingham, Birmingham, Alabama, USA.; 5 Medical Service at Birmingham VA Medical Center, Birmingham, Alabama, USA.; 6 Department of Internal Medicine, University of South Florida, Tampa, Florida, USA.; 7 Brigham and Women’s Hospital, Harvard Medical School, Boston, Massachusetts, USA.; 8 Division of Neonatology, Department of Pediatrics,; 9 Department of Surgery,; 10 Nephrology Research & Training Center, Division of Nephrology, Department of Medicine,; 11 Division of Pulmonary, Allergy and Critical Care Medicine, Department of Medicine, and; 12 Lung Health Center, University of Alabama at Birmingham, Birmingham, Alabama, USA.; 13 Tampa General Hospital, Tampa, Florida, USA.; 14 Department of Cell, Developmental, and Integrative Biology, University of Alabama at Birmingham, Birmingham, Alabama, USA.

**Keywords:** Cardiology, Transplantation, Extracellular matrix, Neutrophils, Organ transplantation

## Abstract

Altered inflammation and tissue remodeling are cardinal features of cardiovascular disease and cardiac transplant rejection. Neutrophils have increasingly been understood to play a critical role in acute rejection and early allograft failure; however, discrete mechanisms that drive this damage remain poorly understood. Herein, we demonstrate that early acute cardiac rejection increases allograft prolyl endopeptidase (PE) in association with de novo production of the neutrophil proinflammatory matrikine proline-glycine-proline (PGP). In a heterotopic murine heart transplant model, PGP production and PE activity were associated with early neutrophil allograft invasion and allograft failure. Pharmacologic inhibition of PE with Z-Pro-prolinal reduced PGP, attenuated early neutrophil graft invasion, and reduced proinflammatory cytokine expression. Importantly, these changes helped preserve allograft rejection-free survival and function. Notably, within 2 independent patient cohorts, both PGP and PE activity were increased among patients with biopsy-proven rejection. The observed induction of PE and matrikine generation provide a link between neutrophilic inflammation and cardiovascular injury, represent a potential target to reduce allogenic immune responses, and uncover a mechanism of cardiovascular disease that has been previously unrecognized to our knowledge.

## Introduction

More than 30 million patients within the United States were diagnosed with a cardiovascular disease by 2018 (1). The combination of longer life expectancies and increased disease prevalence (2) underscores the need to optimize all cardiovascular therapies, including those as limited as heart transplantation. Cardiac allograft rejection is experienced at least once within the first year after transplantation by 20%–50% of patients, with an increased incidence of acute cellular rejection (ACR) occurring within the first 6 months (3). Despite increased morbidity and mortality, there have been limited advancements in the therapies available to treat this feared, early complication.

Neutrophils have previously been shown to play an important role in the initial immune response of ACR. In particular, neutrophils are among the first inflammatory cells to infiltrate transplanted organs in response to ischemia/reperfusion injury and/or production of inflammatory cytokines and damage associated molecular patterns (4). El-Sawy and colleagues previously demonstrated that early neutrophil-mediated tissue damage in a vascularized, heterotopic murine model of cardiac allograft rejection helps promote T cell–mediated rejection (5). In particular, these investigators observed that inhibition of neutrophil allograft infiltration significantly improved the efficacy of T cell costimulatory blockade, leading to improved allograft survival. Separate investigations have also shown neutrophil depletion to slow ACR by attenuating the recruitment of alloreactive memory CD8^+^ T cells (6) and to promote immunosuppression-mediated allograft acceptance, leading to reduced type 1 helper (Th1) cell alloimmunity (7).

Clinically, upregulation of neutrophil adhesion molecule CD11b prior to cardiac transplantation has been shown to predict rejection grade at the time of first endomyocardial biopsy (8), further underscoring the importance of neutrophil activation in early allograft rejection. Hence, unabated neutrophil activation can help establish and orchestrate a more chronic, adaptive allogenic immune response. Taken together, these findings highlight an increased appreciation for neutrophils as early mediators of alloimmunity after organ transplantation (4).

Given the established importance of early neutrophil activation, strategies designed to interrupt neutrophil recruitment in acute rejection may provide therapeutic targets. Extracellular matrix–derived (ECM-derived) chemokines (i.e., *matrikines*) originate from the fragmentation of the ECM proteins (9). Matrikines are implicated in inflammation and immune responses (10), and they can be generated by neutrophil-associated proteases released into the extracellular space during degranulation or exocytosis (11, 12). One such enzyme is the serine protease prolyl endopeptidase (PE) (13). Primarily a cytosolic enzyme (14), release of PE has been described in the sequential production of the matrikine proline-glycine-proline (PGP) (13, 15), a neutrophil chemoattractant (16) and novel biomarker of ACR after heart transplantation (17). PGP generated by the sequential action of MMPs (e.g., MMP9) and PE-mediated degradation of collagen (13, 15) can activate CXCR2 on a variety of cell types (16), and it has been described as a mediator of endothelial dysfunction owing to increased endothelial permeability (18) and endothelin-1 (ET-1) production (17). Hence, in response to allograft injury, PE and associated PGP production may prime the local tissue microenvironment, thereby propagating inflammation.

Given this evidence, we tested the hypothesis that PE promotes allograft neutrophil invasion and mediates acute rejection after heart transplantation. In a heterotopic murine heart transplant model, we observed that early acute cardiac rejection was associated with de novo production of both PGP and increased PE activity. These changes were associated with early neutrophil invasion and allograft failure. Pharmacologic inhibition of PE reduced PGP production, attenuated early neutrophil graft invasion, and reduced proinflammatory cytokine expression. Importantly, these changes helped preserve allograft function and rejection-free survival. Finally, 2 independent patient cohorts demonstrated both increased PGP and PE activity in association with biopsy-proven rejection in comparison with nonrejecting patients. Thus, the following results identify PE activity and possible matrikine generation as a critical pathway impacting early allograft inflammation and provide a therapeutic target to maintain allograft function and survival.

## Results

### Acute allograft rejection in a vascularized mouse model of heart transplantation induced neutrophilic inflammation.

Eight- to ten-week-old, male, BALB/c mice received heterotopic heart transplants from similarly aged BALB/c (isograft) or C57BL/6 (allograft) donor mice. Allografts represented a complete major histocompatibility complex mismatch (MHC *H2^d^* vs. MHC *H2^b^*) and were allowed to undergo acute rejection without immunosuppressive therapies. Mice were sacrificed 3 days after transplant to evaluate early immune responses. Compared with syngeneic isografts, allografts were observed to have marked intramyocardial and perivascular inflammatory cell invasion (Supplemental Figure 1, A–F; supplemental material available online with this article; https://doi.org/10.1172/jci.insight.139687DS1). IHC staining for the neutrophil marker myeloperoxidase (MPO) revealed significant extracellular neutrophil invasion within allografts, whereas little to no staining was visualized within isografts (Supplemental Figure 1, C and F). This neutrophilic response was further quantified by direct neutrophil cell counts, which showed greater neutrophilic graft invasion (Supplemental Figure 1G) as well as marked increases in allograft MPO expression (Supplemental Figure 1H). Together, these findings confirm previous reports of neutrophil activation as an early mediator of acute transplanted organ rejection (4, 5) and underscore neutrophil chemotaxis as a conserved mechanism within the allograft immune response.

### Allograft neutrophilic invasion induced the PGP protease cascade during acute rejection.

**Figure 1:**
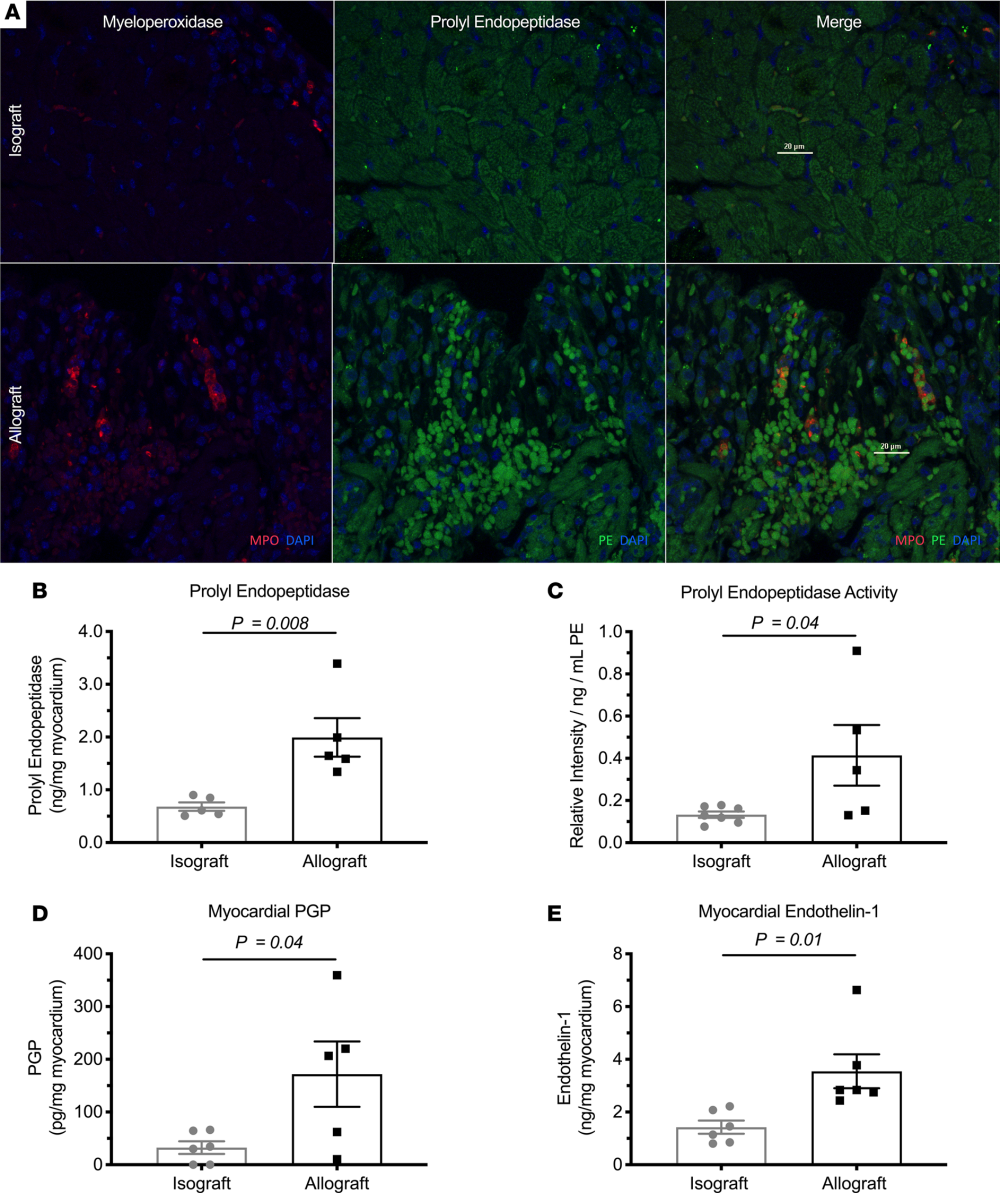
Acute allograft transplant rejection induces graft neutrophil PE and the PGP protease cascade. Eight- to ten-week-old, male, BALB/c mice received heterotopic heart transplants from similarly aged BALB/c (isograft) or C57BL/6 (allograft) donor mice. Mice were sacrificed 3 days after transplant. Among allograft mice, laser confocal microscopy revealed colocalization of PE with the neutrophil marker myeloperoxidase within the extracellular matrix (**A**). This neutrophilic response was further quantified in Supplemental Figure 1. Importantly, both total expression of the PGP-generating metalloprotease PE (**B**) (*n* = 5) and PE activity (**C**) (*n* = 7 and *n* = 5, respectively) were increased within allograft hearts and associated with de novo PGP production (**D**) (*n* = 5). Finally, increased myocardial PGP production was positively associated with ET-1 (**E**) (*n* = 6), an established marker of PGP activity. Results presented as mean ± SE. Where indicated, *n* represents animals/group. Student’s unpaired *t* test was used for each comparison. PE, prolyl endopeptidase; PGP, proline-glycine-proline; ET-1, endothelin-1.

MMP9 has previously been established as a mediator of myocardial transplant rejection (17, 19) and key catalyst for the production of PGP (15). Experimentally, allograft transplants were observed to have increased myocardial MMP9 expression and enzymatic activity (Supplemental Figure 1, I and J). Allograft rejection was also associated with increased expression of total PE (Figure 1B and Supplemental Figure 2) and increased enzymatic PE activity (Figure 1C). Confocal microscopy revealed colocalization of PE with invading neutrophils within the extracellular space (Figure 1A), highlighting neutrophils as an inducible source of PE and MMP9 in acute rejection. Importantly, these observations were associated with de novo generation of PGP (Figure 1D). ET-1, a marker of endothelial injury and PGP activity (17), was also significantly increased (Figure 1E) within allografts when compared with isografts. These results, in combination with allograft neutrophil chemotaxis (Supplemental Figure 1), highlight induction of the PGP protease cascade by neutrophils and suggest active PGP signaling in response to early acute myocardial rejection.

### PE inhibition attenuated allograft PGP activity and neutrophil chemotaxis.

**Figure 2:**
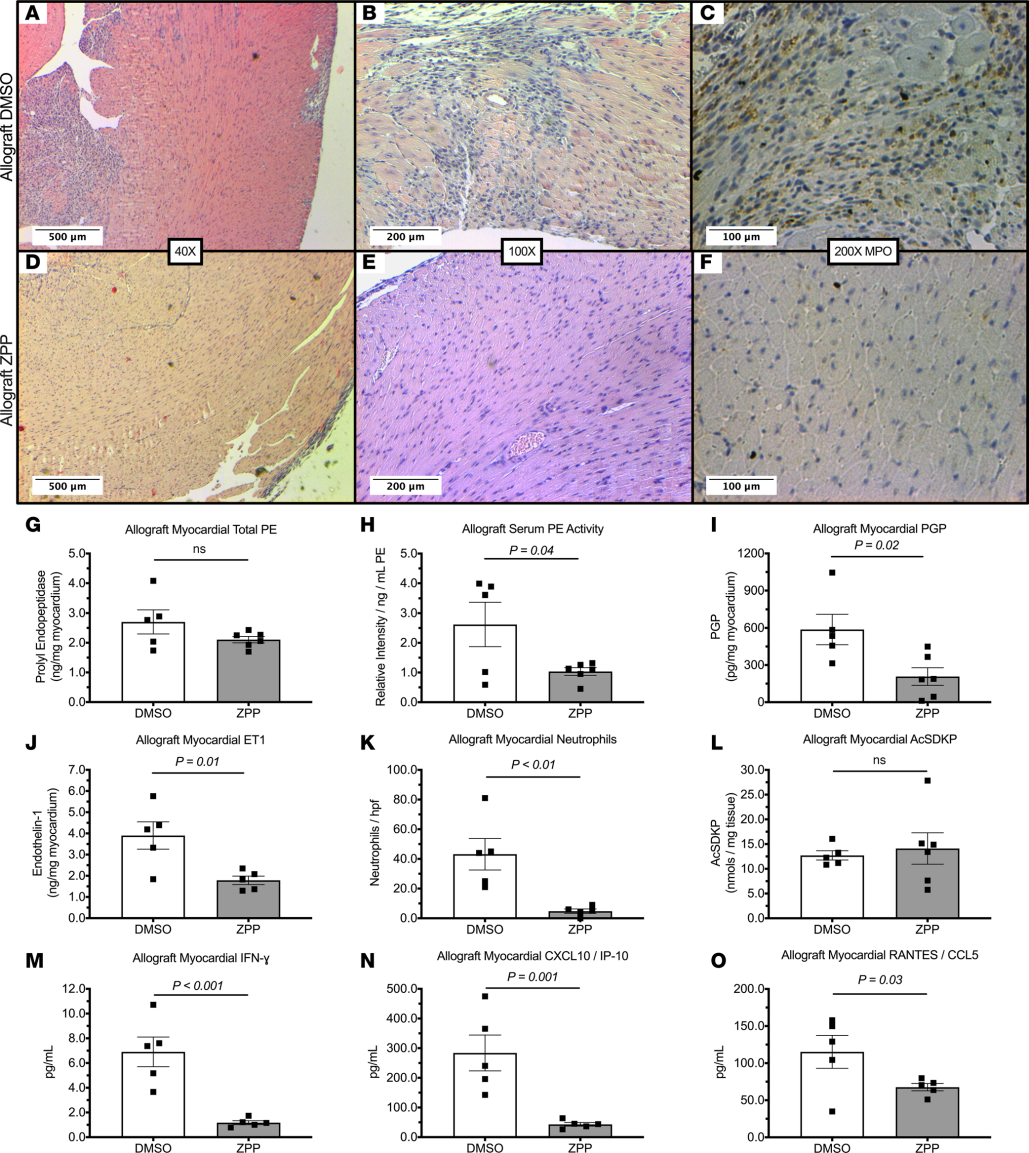
PE inhibition attenuates allograft inflammatory response. Allograft mice were treated with ZPP (10 mg/kg i.p.) or a 2% DMSO vehicle control. (**A–F**) illustrates representative images of H&E and IHC staining. Compared with controls (**A–C**), ZPP reduced allograft inflammatory cell invasion (**D** and **E**) and MPO expression (**F**). Although total allograft PE did not change (**G**) (*n* = 5 animals/group), ZPP administration significantly reduced serum PE activity (**H**) (*n* = 5 DMSO and *n* = 6 ZPP). Importantly, ZPP significantly reduced allograft PGP (**I**) (*n* = 5 DMSO and *n* = 6 ZPP) and ET-1 (**J**) (*n* = 5 animals/group). This observation was associated with reduced intragraft neutrophil invasion (**K**) (*n* = 5 animals/group). In contrast, concentrations of the antifibrotic peptide AcSDKP were not altered by the administration of ZPP (**L**) (*n* = 5 DMSO and *n* = 6 ZPP animals/group). Importantly, ZPP attenuated the expression of allograft IFN-γ and associated leukocyte chemoattractants CXCL10 and RANTES (**M–O**) (*n* = 5 DMSO and *n* = 7 ZPP). Results presented as mean ± SE. Student’s unpaired *t* test was used for each comparison. PE, prolyl endopeptidase; PGP, proline-glycine-proline; ET-1, endothelin-1; ZPP, Z-Pro-Prolinal; MPO, myeloperoxidase; AcSDKP, acetyl-N-Ser-Asp-Lys-Pro.

Age-matched allograft transplant mice were treated with the PE inhibitor Z-Pro-prolinal (ZPP; 10 mg/kg i.p., daily) (20) or a 2% DMSO vehicle control. Fitting with our hypothesis, ZPP reduced allograft inflammatory cell invasion, whereas DMSO-treated allograft controls continued to have a robust inflammatory response (Figure 2, A–F). As expected, ZPP did not affect allograft total PE expression (Figure 2G), but it significantly reduced allograft serum PE activity compared with DMSO-treated allograft controls (Figure 2H). More importantly, ZPP administration significantly reduced allograft PGP and ET-1 concentrations (Figure 2, I and J) to levels comparable with syngeneic isograft mice (Figure 1). As expected, diminished PGP production was associated with decreased total neutrophil graft invasion (Figure 2, F and K). Interestingly, local concentrations of the antifibrotic peptide acetyl-N-Ser-Asp-Lys-Pro (AcSDKP) were unchanged with short-term PE inhibition with ZPP (Figure 2L), thereby preserving an established inhibitor of organ fibrosis. Together, these results identify early PE activity as an inducible, proinflammatory mediator of neutrophil trafficking in acute rejection.

### PE inhibition attenuated allograft inflammatory response.

Administration of ZPP ablated the Th1-promoting cytokine IFN-γ within allografts on day 3 after transplantation (Figure 2M). Additionally, ZPP reduced the IFN-γ inducible cytokines T cell chemoattractants chemokine IFN-γ–inducible protein 10 (CXCL10; also known as IP-10; Figure 2N) and chemokine regulated on activation, normal T cell expressed and secreted (RANTES; also known as CCL5; Figure 2O). In contrast, the Th2-promoting cytokine IL-4 was observed to have a nonsignificant reduction within ZPP-treated allograft mice (Supplemental Figure 3A). Together, these observations suggest a reduced Th1-mediated adaptive immune response in association with reduced neutrophilic inflammation. Interestingly, administration of ZPP also significantly attenuated the innate immune mediator IL-1β, with an associated trend toward reduced IL-6 (Supplemental Figure 3, B and C). As crucial promoters of innate and adaptive cellular immunity, these results highlight PE generation of PGP and subsequent neutrophil activation as potential regulators of early allograft immune response.

### PE inhibition preserved allograft function and improved rejection-free survival.

**Figure 3:**
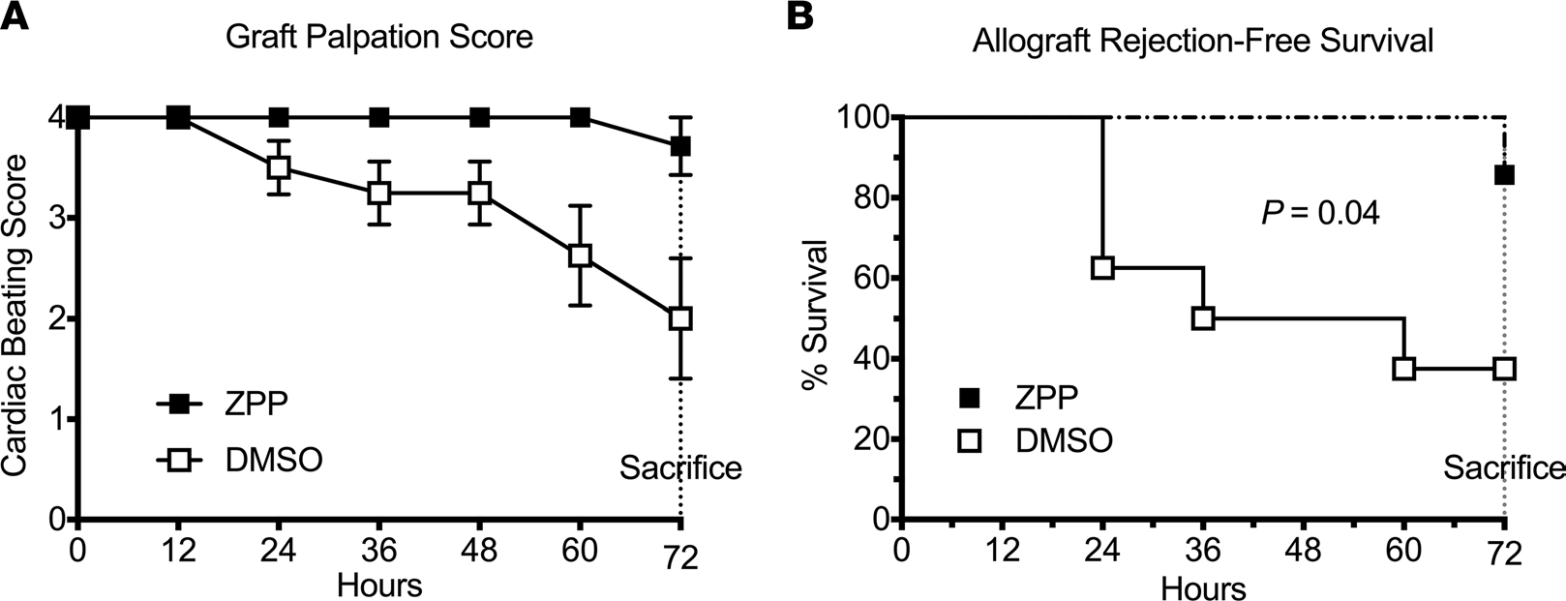
PE inhibition preserves allograft function and improves rejection-free survival. Allograft viability was assessed by cardiac graft beating score (**A**). ZPP improved allograft rejection-free survival on day 3 with a median DMSO survival of 48% (**B**) (*n* = 8 DMSO-treated and 7 ZPP-treated animals). Log rank test was used for survival analysis and Student’s unpaired *t* test was used for the comparison. Results presented as mean ± SE. PE, prolyl endopeptidase.

In light of our findings, we assessed the relative function and viability of allografts treated with either ZPP or DMSO. As a marker of rejection severity and graft function, allograft cardiac beating scores (Figure 3A) were assessed in the postoperative period. Compared with DMSO-treated allografts, ZPP administration preserved allograft function. Importantly, ZPP also improved allograft rejection-free survival on day 3 with a median DMSO survival of 48% (Figure 3B). Although the mechanisms of acute rejection remain to be better elucidated (including potential roles for innate lymphoid cells), our ability to attenuate proinflammatory mediators of rejection and improve allograft survival via a selective protease inhibitor provides a paradigm for immunomodulatory therapies.

### The PGP pathway was active among cardiac transplant patients with rejection.

**Figure 4:**
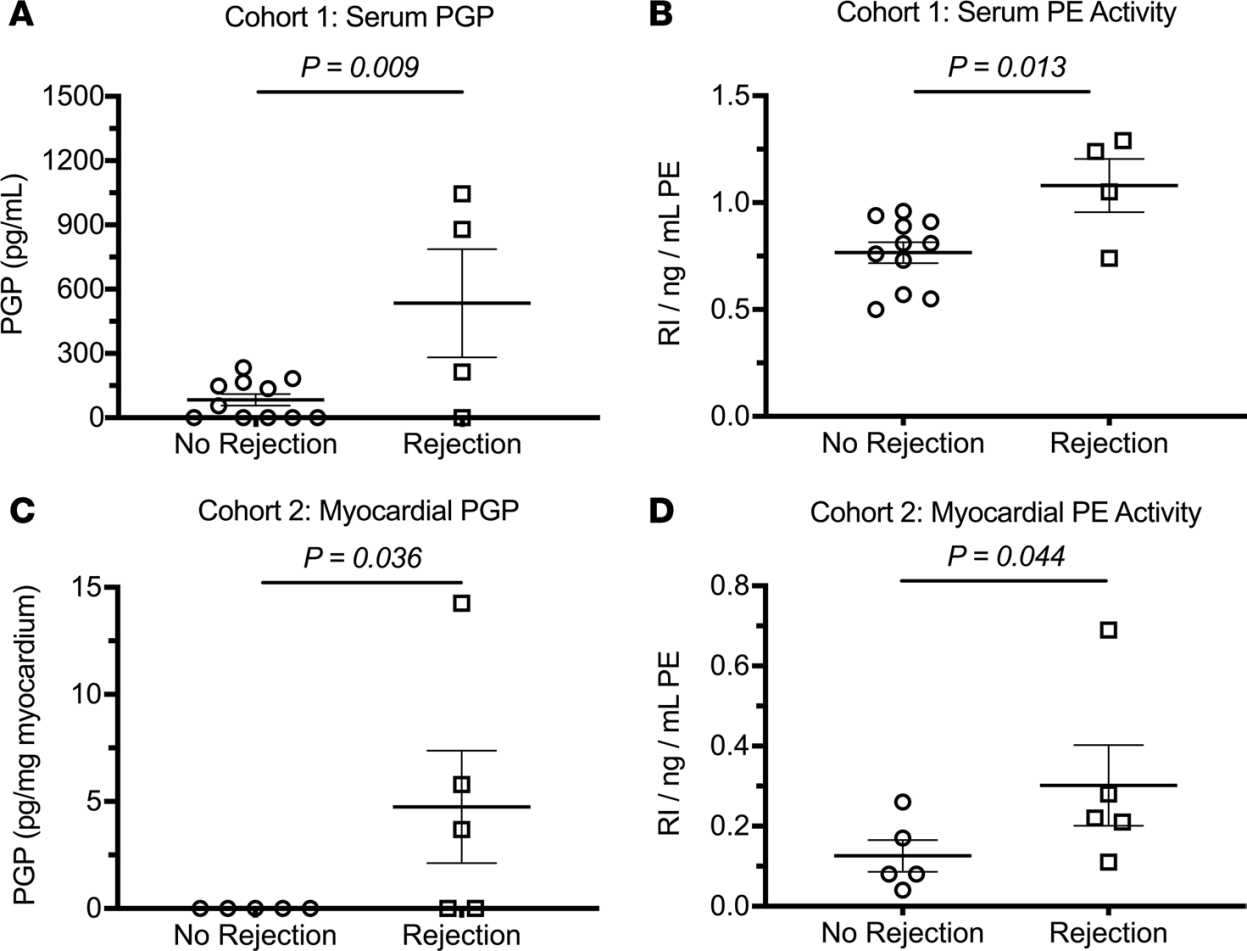
Increased PGP and PE activity are associated with biopsy-proven ACR among 2 independent cohorts of patients with cardiac transplants. (**A**) PGP, measured from the left main coronary artery, was increased among patients with cardiac transplants with ACR (*n* = 4) compared with those without ACR (*n* = 11). (**B**) Within the same patient cohort, coronary serum PE activity was increased in association with rejection. Myocardial biopsies from an independent patient cohort showed greater myocardial biopsy PGP (**C**) and PE activity (**D**) among patients with ACR (*n* = 5) compared with those without ACR (*n* = 5). Results presented as mean ± SE. Student’s unpaired *t* test was used for comparisons in **A** and **B**. Mann-Whitney *U* test was used for comparisons in **C** and **D**. PGP, proline-glycine-proline; PE, prolyl endopeptidase; ACR, acute cellular rejection.

Importantly, results from 2 independent heart transplant patient cohorts validated our experimental observations (Table 1). Patients within cohort 1 underwent surveillance left and right heart catheterization with myocardial biopsy. Biopsies and coronary serum were collected during the same procedure from 12 patients undergoing catheterizations at 6 weeks and 1 year after transplantation (*n* = 15 unduplicated samples at unique time points with variable rejection status). Compared with patients without rejection (*n* = 8), patients with rejection (*n* = 4) had coronary PGP levels that were 6-fold higher compared with patients without evidence of ACR on simultaneously collected biopsies (Figure 4A). Cohort 1 patients were also observed to have increased coronary serum PE activity (Figure 4B). To further validate these observations, remnant transplant myocardial biopsy samples from a second independent patient cohort were obtained from a separate institution (Cohort 2). Fitting with our original observations, biopsy samples with ACR had increased myocardial tissue PGP and significantly increased myocardial PE activity compared with those free of rejection (Figure 4, C and D). Together, these results associate PGP with acute cardiac rejection within 2 independent patient cohorts and highlight PE as a possible modifiable target to reduce cardiac inflammation.

## Discussion

Recent investigations have implicated neutrophil activation as a direct mediator of early cardiac allograft inflammation and failure (4, 5, 21). Despite this knowledge, discrete mechanisms that drive neutrophil invasion and tissue damage remain poorly understood. Accordingly, this investigation studied the serine protease PE as a mechanism of allograft rejection and cardiovascular injury. The findings of this study are as follows: (a) PE was upregulated with acute cardiac rejection in a vascularized heterotopic mouse model; (b) PE activity was associated with de novo production of the neutrophil proinflammatory matrikine PGP; (c) inhibition of PE attenuated early neutrophil graft invasion and inflammation; (d) PE inhibition prolonged rejection-free graft survival and function in a model of acute cardiac rejection; and (e) PE activity and PGP were associated with biopsy-proven rejection among 2 independent cohorts of patients with heart transplants. Although preliminary, these results underscore PE activity and potential matrikine generation as mechanisms of acute cardiac injury and cardiovascular inflammation.

Previous investigations have identified early neutrophil recruitment and activation via CXCR2 as a driver for accelerated cardiac graft rejection (5). Interestingly, pharmacologic inhibition of CXCR1/2 with repertaxin has been shown to improve clinical outcomes among patients receiving human pancreatic islet cell transplants through a proposed mechanism of reduced neutrophil and natural killer T cell recruitment (22). Our findings confirm previous reports of neutrophil activation as an early mediator of acute transplanted organ rejection (4) and underscore neutrophil chemotaxis as a conserved mechanism within the allograft immune response. Furthermore, inhibition of PE reduces PGP, an established CXCR2 ligand and neutrophil chemoattractant (16), in response to early cardiac allograft rejection. These observations expand our understanding of how CXCR2 ligands such as PGP potentiate neutrophilic inflammation in acute rejection. However, future investigations are needed to further validate a causal role for PGP (separate from PE activity) in promoting rejection and delineate the predominant ECM source for PGP generation.

Inhibition of PE reduced the expression of proinflammatory cytokines, notably IFN-γ and IL-1β, both of which are known to help orchestrate a more robust inflammatory response and transition to adaptive immunity (Figure 2). Classically, the allogenic immune response results in activation of T cells in part by upregulation of Class II MHC antigens and associated cytokines (23). Transition from an innate to adaptive cellular immune response, due in part to early neutrophilic activation, may potentially be promoted by PE activity. Although the current investigation is limited in its focus on matrikine-generated and protease-generated neutrophil chemotaxis, the observed reductions of Th1 cytokines highlight the need for additional work to study the link between PE and adaptive immunity. Most notably, experimental studies with longer time points of allograft survival (with and without immunosuppressive therapies) would help delineate the potential benefit of early PE inhibition on other inflammatory mediators of acute and chronic rejection. In addition, our pharmacologic use of ZPP to diminish PE activity may have unknown off-target and/or pleiotropic effects not observed by our current experimental approach. Although previous studies have clearly demonstrated the efficacy of using ZPP to inhibit PE and PGP production (24), additional studies are nonetheless important to better describe the pharmacologic effect of this drug and verify a causal effect of PGP as a CXCR2 ligand.

Timing and duration of PE inhibition after an inflammatory injury is also an important issue for consideration. PE has been well described as a postproline-cleaving enzyme with high affinity to the C-terminus of prolines within short peptides less than 30 amino acids in length (25). In addition to PGP, extracellular activity of PE has been linked to the release of the antifibrotic tetrapeptide AcSDKP from thymosin-β_4_ (26, 27). Importantly, our short-term inhibition of PE (3 days) failed to change tissue concentrations of AcSDKP (Figure 2L), thereby preserving an established inhibitor of organ fibrosis. Although future studies are necessary to delineate the pathophysiology of ECM remodeling by PE, the current findings highlight a potentially delicate homeostasis regulated by matrikines in response to injury.

To date, few novel therapies have emerged to reduce cardiovascular inflammation or ACR. The current results support a direct role for matrikine regulation of neutrophil-mediated allograft rejection. Clinically, our results link PE with acute allograft rejection within 2 independent patient cohorts (Figure 4). Although we have previously described PGP as a potential circulating biomarker of rejection (17), the current investigation extends this work by showing that the PGP enzymatic pathway is locally active within allografts and directly associated with neutrophil invasion. Furthermore, our results support PE as a possible modifiable target of early acute cardiac rejection. Although our investigation is not without limitations (most notably small patient cohorts), future trials in cardiovascular disease may benefit from incorporating PGP as a proinflammatory biomarker and targeting PE to reduce myocardial inflammation. Together, our observations highlight a therapeutic target separate from classical immunosuppressive agents that may improve solid organ transplant function and viability.

More importantly, the current work likely has tremendous implications to therapeutic approaches for the treatment of more common cardiovascular disorders. Induction of PE and PGP peptides is not likely limited to alloimmune responses and may help orchestrate innate immune responses within an array of vascular-associated diseases such as heart failure, pulmonary hypertension, atherosclerosis, and/or myocardial infarction. Altogether, our findings draw attention to matrikine biology as an underappreciated regulator of cardiovascular inflammation and disease.

## Methods

### Patient samples

#### Patient demographics.

Patient demographics are documented in Table 1. Patients who had orthotopic heart transplants were recruited from the University of Alabama at Birmingham (UAB; *n* = 12) and Tampa General Hospital/University of South Florida (USF; *n* = 10). Patient demographics showed no significant differences in age, sex, or immunosuppressive medications.

#### Myocardial and blood sample preparation.

Right ventricular biopsies were collected from 2 independent patient cohorts at UAB and Tampa General Hospital/USF hospitals. Biopsies were collected during right heart catheterization as standard of care for cardiac rejection surveillance as previously described (17). A total of 22 patients were enrolled between both sites. At UAB, left main coronary artery serum was collected from 12 patients at the same time as biopsy sampling. Samples were collected at 6 weeks and 1 year after transplantation (*n* = 15 unduplicated samples at unique time points with variable rejection status). Independent pathologists reviewed specimens for evidence of rejection according to established International Society for Heart and Lung Transplantation (ISHLT) guidelines (28). At USF, remnant biopsy samples were flash frozen in liquid nitrogen and processed for ELISA and/or mass spectrometry. Total protein was isolated by tissue homogenization in a lysis buffer of PBS with protease inhibitor cocktail (MilliporeSigma, catalog P8340, 1:100 dilution). Samples from both cohorts were stored at –80°C prior to experimentation.

### Animal model

#### Murine heterotopic heart transplant model.

Eight- to ten-week-old, male, BALB/c (MHC *H2^d^*) and C57BL/6 (MHC *H2^b^*) mice were obtained from The Jackson Laboratory. Vascularized heterotopic cardiac transplantation was performed as described by Corry et al. (29). Briefly, mice were anesthetized with ketamine (80–100 mg/kg s.c.) and xylazine (5–10 mg/kg s.c.) and used as either donors or recipients. Buprenorphine (0.05-0.1 mg/kg s.c.) was provided for analgesia. Donor mice were anticoagulated by 1-time injection of heparin (250 IU) into the inferior vena cava immediately before harvesting the heart. Donor hearts were anastomosed to recipient aorta and vena cava using microsurgical procedures. Syngeneic isografts (BALB/c × BALB/c) and haplotype mismatched allografts (C57BL/6 × BALB/c) were monitored twice daily by palpation through the abdominal wall. Prior to sacrifice on postoperative day 3, graft function was assessed by palpation and confirmed by echocardiography. Standard palpation scoring on a scale of 0–4 was used to assess graft function with 4 representing normal amplitude and frequency and 0 representing a nonbeating, rejected graft (30). Graft failure was considered if there was no palpable heart beat and/or cardiac arrest visualized on echocardiography. Staging of rejection was confirmed at the time of sacrifice. All mice were sacrificed on day 3 with collection of serum and graft tissue for biochemical or histologic analysis. Where indicated, allograft transplant recipient mice were treated immediately after surgery and daily thereafter with the PE inhibitor ZPP (10 mg/kg i.p., K_i_ = 1 nM, MilliporeSigma) or a 2% DMSO vehicle control. No immunosuppressive agents were used throughout the study.

#### Murine heterotopic allograft echocardiography.

Vevo 3100 (VisualSonics Inc.) in vivo imaging system was used to perform echocardiography equipped with probes of up to 40 MHz and a resolution of 30 μm. Transplant recipient mice were anesthetized with 1.5%–2% isoflurane in an oxygen mix. Heart rate (>400 beats per minute), respiratory rate, and body temperature (35°C–37°C) were continuously monitored throughout the procedure to ensure an adequate depth of anesthesia. Heterotopic grafts were identified and imaged within the abdomen. Mmode and 2-dimensional grayscale echocardiographic images were acquired from long-axis and short-axis views. Graft heart rates were estimated from peak-to-peak measurements on Mmode images.

#### Murine tissue preparation.

Murine blood samples were centrifuged at 1200 *g* for 15 minutes, and serum was aspirated and stored at –80°C. Murine myocardial tissues assessed for ELISA were removed immediately after sacrifice and snap-frozen in liquid nitrogen. The tissue was lysed with either RIPA lysis buffer (Santa Cruz Biotechnology) or PBS containing protease inhibitor cocktail (MilliporeSigma, catalog P8340, 1:100 dilution) prior to homogenization. Lysates were clarified by centrifugation at 14,000*g* for 10 minutes, and protein concentration as determined by BCA assay (Bio-Rad).

### In vitro experiments

#### ELISA.

MPO, ET-1, and MMP9 concentrations for myocardial tissue lysates were measured using commercially available ELISA kits and protocols from R&D Systems. Total PE and AcSDKP concentrations for serum and myocardial tissue lysates were similarly measured with mouse and/or human PE ELISA kits from My Bio Source. IFN-γ, CXCL10, and RANTES were all measured by multiplex ELISA panel (Mouse Cytokine/Chemokine ProcartaPlex Panel 1, Invitrogen, catalog EPX260-26088-901). Where indicated, results from tissue lysates were normalized to sample total protein concentrations.

#### Murine MMP9 zymography.

Tissue MMP9 activity was assessed by zymography as previously described (31). Briefly, samples were diluted in nonreducing sample buffer, and 10 μg of sample was added to each lane of a 7.5% SDS-polyacrylamide gel with 1.0 mg/mL porcine skin gelatin. All samples are electrophoresed at 45V for 5 hours at 4°C. After electrophoresis, gels were washed in 2.5% Triton X-100 for 30 minutes at 25°C and then incubated in 50 mM Tris and 5.0 mM CaCl (pH = 8.0) for 16 hours at 37°C. Gels were stained in 0.05% Coomassie blue for 30 minutes and subsequently distained in acetic acid and methanol for optimal exposure. Higher MW bands on gelatin zymograms represented MMP9 complexes. ImageJ (NIH) software was used to quantify relative MMP9 intensity.

#### PE activity assays.

Myocardial and serum PE activity was determined as previously described (12) by incubating samples with a PE-specific substrate, 1 mM SUC-Gly-Pro-AMC (N-succinyl-glycine-proline-7-amido-4-methyl-coumarin) at 37°C. Cleavage of AMC by PE was detected using a fluorometer using excitation and emission wavelengths of 380 nm and 460 nm, respectively. Results are presented as a ratio of measured activity to total measured PE.

#### Electrospray ionization-liquid chromatography–tandem mass spectrometry.

Serum and homogenized myocardial samples processed in PBS with protease inhibitor cocktail (MilliporeSigma, catalog P8340, 1:100 dilution) were filtered through a Millipore 10,000–MW cutoff centrifugal filter followed by washing with 30 μL of 1 mM HCl. PGP was measured using a MDS Sciex API-4000 spectrometer (Applied Biosystems) equipped with a Shimadzu HPLC. HPLC was performed using a 2.0_150-mm Jupiter 4 μm Proteo column (Phenomenex) with A: 0.1% HCOOH and B: MeCN plus 0.1% HCOOH: 0–0.5 minutes 5% buffer B/95% buffer A, then increased over 0.5–2.5 minutes to 100% buffer B/0% buffer A. Background was removed by flushing with 100% isopropanol/0.1% formic acid. Positive electrospray mass transition was at 270–173 for PGP.

#### Histology and IHC.

All myocardial tissue specimens were fixed with 10% neutral buffered formalin (Fisher Scientific) at room temperature for 24 hours. Samples were then dehydrated and paraffin embedded prior to serial 5 μm thick sectioning. Sections were finally floated onto gelatin-coated charged glass slides (Super-Frost Plus, Fisher Scientific) and dried overnight at 60°C. All sections were deparaffinized and hydrated using graded concentrations of ethanol to deionized water. All sections for immune cell infiltration were stained with hematoxylin for 7 minutes followed by eosin for 1 minute. The sections were then dehydrated with gradient alcohol, cleared in xylene for 10 minutes, and mounted with neutral balsam.

Tissue sections for IHC were subjected to antigen retrieval by 0.01 M Tris-1 mM EDTA buffer (pH 9) in pressure cooker for 5 minutes (buffer preheated with the steam setting for 10 minutes). After antigen retrieval, all sections were washed gently in deionized water, then transferred in to 0.05 M Tris-based solution in 0.15 M NaCl with 0.1% v/v Triton-X-100, pH 7.6 (TBST). Endogenous peroxidase was blocked with 3% hydrogen peroxide for 20 minutes. To reduce further nonspecific background staining, slides were incubated with 3% normal goat serum or 3% normal rabbit serum for 45 minutes (MilliporeSigma) at room temperature. All slides then were incubated at 4°C overnight with the antibodies (MPO, rabbit IgG, 1:100, catalog PA5-16672, Invitrogen; or PE, goat IgG, 1:100, ab110857, Abcam). Negative controls for PE stains were produced using a goat IgG, polyclonal isotype control antibody (ab37373, Abcam). Negative controls for all other stains were produced by eliminating the primary antibodies from the diluents. After washing with TBST, slides were incubated with the goat anti–rabbit IgG H&L (HRP) (1:1000, ab6721, Abcam) or rabbit anti–goat IgG H&L (HRP) (1:500, ab97100, Abcam) 45 minutes at room temperature. DAB (Scy Tek Laboratories) was used as the chromogen and hematoxylin (no. 7211, Richard-Allen Scientific) as the counterstain. Histopathological changes in the tissue were observed and photographed with a light microscope (Reichert Biostar). Neutrophil cell counts were obtained by counting positive MPO staining cells within a high-power field (original magnification, ×20).

#### Confocal microscopy.

Tissue sections for immunofluorescence microscopy underwent antigen retrieval as described above. Primary antibodies for PE (Abcam 246978, 1:200 in PHEM buffer, Alexa 488) and MPO (Abcam 208670, 1:200 in PHEM, Alexa 594) were directly conjugated with fluorophores using an antibody labeling kit (Novus Biologicals 322-0030 and 335-0030) prior to immunostaining. All slides were incubated at 4°C overnight. After washing in PBST (0.2% Tween-20), a DAPI stain (100 ng/mL) was added. Slides were imaged using a Nikon A1R laser confocal microscope and Nis Elements 5.0 acquisition software with the same settings (objective: Plan Fluor 40× Oil DIC H N2; resolution: 1024 × 1024 pixels).

### Statistics

All results are displayed as mean ± SEM. The Student’s unpaired 2-tailed *t* test or Mann Whitney *U* test was used for comparisons of the mean values of 2 different samples where indicated. A *P* value less than or equal to 0.05 was considered significant. A log rank test was used for survival analysis of allograft heart transplants. All statistical analyses were performed using GraphPad Prism software.

### Study approval

Human and animal studies were approved by the appropriate institutional review boards. Specifically, all animal studies were approved by the UAB Institutional Animal Care and Use Committee (protocol no. 21483). The care of all animals involved in this study was in accordance with UAB institutional guidelines. Regarding human studies, approval was granted by the UAB (IRB 00000726) and USF IRBs (IRB PR00034027). Written informed consent was received from all participants prior to inclusion in the study. All human and animal studies were performed in accordance with relevant guidelines and regulations.

## Author contributions

GAP, NSS, CVL, CS, LG, CM, LV, JL, DX, XX, JEB, and AG helped design and perform the experimental studies. GAP, NSS, SK, MB, CS, JL, JT, ML, and AG helped design, collect and process clinical samples. JT, ML, NSS, SK, and MB managed all patients. GAP, CM, LG, JFG, and CS designed and managed all experimental animal surgeries. DX, JMW, CVL, JEB, and AG provided critical feedback to investigational aims. GAP wrote the manuscript with substantial editing from AG and JEB.

## Supplementary Material

Supplemental data

## Figures and Tables

**Table 1:**
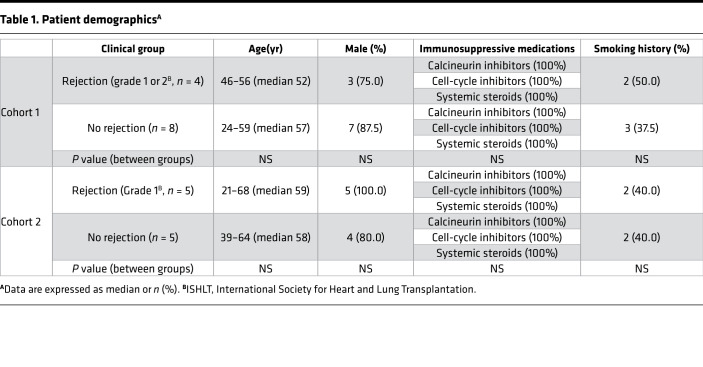
Patient demographics^A^

## Version 1 02/11/2021

In-Press Preview

## Version 2 03/22/2021

Electronic publication
