# Supplementary material for: Prolyl endopeptidase contributes to early neutrophilic inflammation in acute myocardial transplant rejection
Source: JCI Insight. 2021 Mar 22;6(6):e139687. doi: 10.1172/jci.insight.139687 (PMC8026194; doi:10.1172/jci.insight.139687)
Supplement: Supplemental data [file jciinsight-6-139687-s077.pdf]

Supplementary Information:

Prolyl Endopeptidase contributes to early neutrophilic inflammation in acute cardiac transplant rejection

Gregory A. Payne MD, PhD[1-5]*, Nirmal S. Sharma MD[6,14], Charitharth V. Lal MD[4,7], Chunyan Song[1], Lingling Guo MD[8,9], Camilla Margaroli PhD[4,10], Liliana Viera[4,10,11], Siva Kumar MD[6,13], Jindong Li[4,10], Dongqi Xing MD, PhD[2,4,10], Melanie Bosley[13], Xin Xu MD, PhD[4,10], J. Michael Wells MD[4,5,10,11], James F. George PhD[8,9], Jose Tallaj MD[1,3], Massoud Leesar MD[1,3], J. Edwin Blalock PhD[2,4,10-12†] and Amit Gaggar MD, PhD[2,4,5,10-12†]

[1]Division of Cardiovascular Disease, Department of Medicine, University of Alabama at Birmingham, Birmingham, AL
[2]Vascular Biology and Hypertension Program, University of Alabama at Birmingham, Birmingham, AL
[3]Comprehensive Cardiovascular Center, University of Alabama at Birmingham, Birmingham, AL
[4]Program in Protease and Matrix Biology, University of Alabama at Birmingham, Birmingham, AL
[5]Medical Service at Birmingham VA Medical Center, Birmingham, AL
[6]Department of Internal Medicine, University of South Florida, Tampa, FL
[7]Division of Neonatology, Department of Pediatrics, University of Alabama at Birmingham, Birmingham, AL
[8]Department of Surgery, University of Alabama at Birmingham, Birmingham, AL
[9]Nephrology Research & Training Center, Division of Nephrology, Department of Medicine, University of Alabama at Birmingham, Birmingham, AL
[10]Division of Pulmonary, Allergy & Critical Care Medicine, Department of Medicine, University of Alabama at Birmingham, Birmingham, AL
[11]Lung Health Center, University of Alabama at Birmingham, Birmingham, AL
[12]Department of Cell, Developmental, and Integrative Biology, University of Alabama at Birmingham, Birmingham, AL
[13]Tampa General Hospital, Tampa, FL
[14]Brigham and Women's Hospital, Harvard Medical School, Boston, MA
**denotes corresponding authors*
†denotes equal contribution

Conflict of Interest: The authors have declared that no conflict of interest exists

Corresponding Author

Gregory A. Payne MD PhD
Zeigler Research Building 531
1720 2nd Avenue South
Birmingham, AL 35294
gpayne@uabmc.edu

SUPPLEMENTAL FIGURE LEGENDS

Supplemental Figure 1. Acute allograft transplant rejection induces neutrophilic inflammation. 8-10 week-old, male, BALB/cj mice received heterotopic heart transplants from similarly aged BALB/cj (isograft) or C57BL/6j (allograft) donor mice. Mice were sacrificed 3 days post-transplant. **A-F** illustrates representative images of hematoxylin and eosin and immunohistochemical staining. Compared to syngeneic isografts (**A-C**), allografts were observed to have marked intramyocardial and perivascular inflammatory cell invasion (**D and E**). Staining for the neutrophil marker MPO revealed significant extracellular neutrophil invasion within allografts (**F**) in comparison to isografts (**C**). This neutrophilic response was further quantified by direct neutrophil cell counts (**G**, *n* = 4), as well as direct measurement of allograft MPO expression by ELISA (**H**, *n* = 5). In addition to induction of PE expression (**Figure 1**), acute rejection within allografts caused a marked increase in total matrix metalloprotease (MMP) 9 expression (**I**, *n* = 7) and activity (**J**, *n* = 6). Together, these findings confirm a neutrophilic response and induction of MMP9 (a key protease associated with PGP generation) during acute allograft rejection. Results presented as mean ± SE. Where indicated, *n* represents animals/group. Student's unpaired t-test was used for each comparison.

Supplemental Figure 2. Acute allograft transplant rejection induces local PE expression. 8-10 week-old, male, BALB/cj mice received heterotopic heart transplants from similarly aged BALB/cj (isograft) or C57BL/6j (allograft) donor mice. Mice were sacrificed 3 days post-transplant. **A-F** illustrates representative images of myocardial tissue sections immunohistochemically stained for PE or an isotype control. Compared to syngeneic isografts (**A and B**), allografts were observed to have significant immune cell infiltration that was associated with a marked increase in extracellular PE expression (**D and E**).

Supplemental Figure 3. PE inhibition attenuates allograft inflammatory response. Allograft mice were treated with Z-Pro-Prolinal (ZPP, 10mg/kg i.p.) or a 2% DMSO vehicle control. Compared to DMSO-treated mice, ZPP reduced key pro-inflammatory mediators (**Figure 2**). The Th2 promoting cytokine IL-4 showed a non-significant reduction with ZPP (**A**). Interestingly, the innate cytokine IL-1β was also significantly reduced (**B**) and associated with a trend towards reduced IL-6 production (**C**). *n* = 5 animals/group. Results presented as mean ± SE. Student's unpaired t-test was used for each comparison.

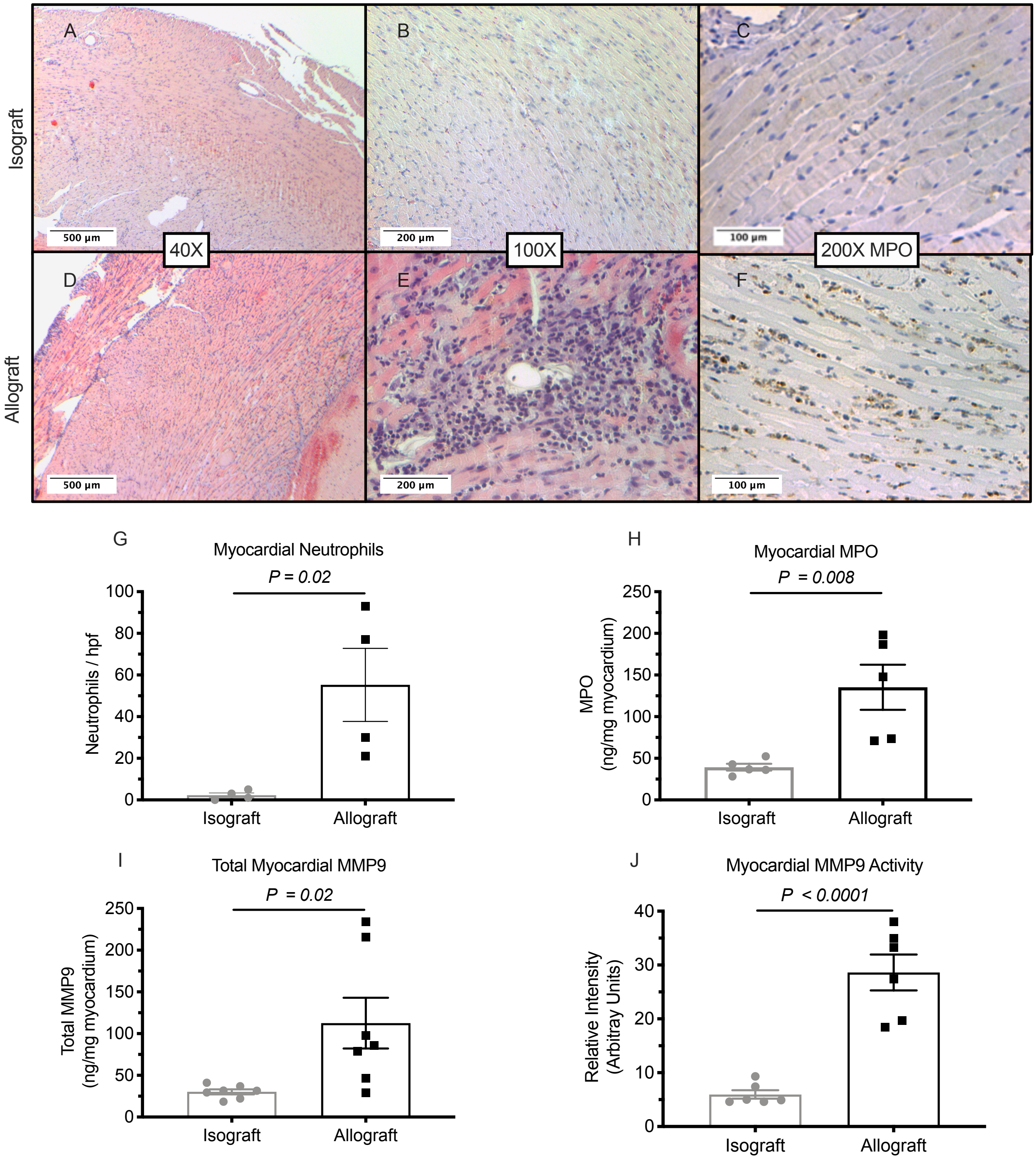

Supplemental Figure 1. Acute allograft transplant rejection induces neutrophilic inflammation. 8-10 week-old, male, BALB/cj mice received heterotopic heart transplants from similarly aged BALB/cj (isograft) or C57BL/6j (allograft) donor mice. Mice were sacrificed 3 days post-transplant. **A-F** illustrates representative images of hematoxylin and eosin and immunohistochemical staining. Compared to syngeneic isografts (**A-C**), allografts were observed to have marked intramyocardial and perivascular inflammatory cell invasion (**D and E**). Staining for the neutrophil marker MPO revealed significant extracellular neutrophil invasion within allografts (**F**) in comparison to isografts (**C**). This neutrophilic response was further quantified by direct neutrophil cell counts (**G**, n = 4), as well as direct measurement of allograft MPO expression by ELISA (**H**, n = 5). In addition to induction of PE expression (**Figure 1**), acute rejection within allografts caused a marked increase in total matrix metalloprotease (MMP) 9 expression (**I**, n = 7) and activity (**J**, n = 6). Together, these findings confirm a neutrophilic response and induction of MMP9 (a key protease associated with PGP generation) during acute allograft rejection. Results presented as mean ± SE. Where indicated, n represents animals/group. Student's unpaired t-test was used for each comparison.

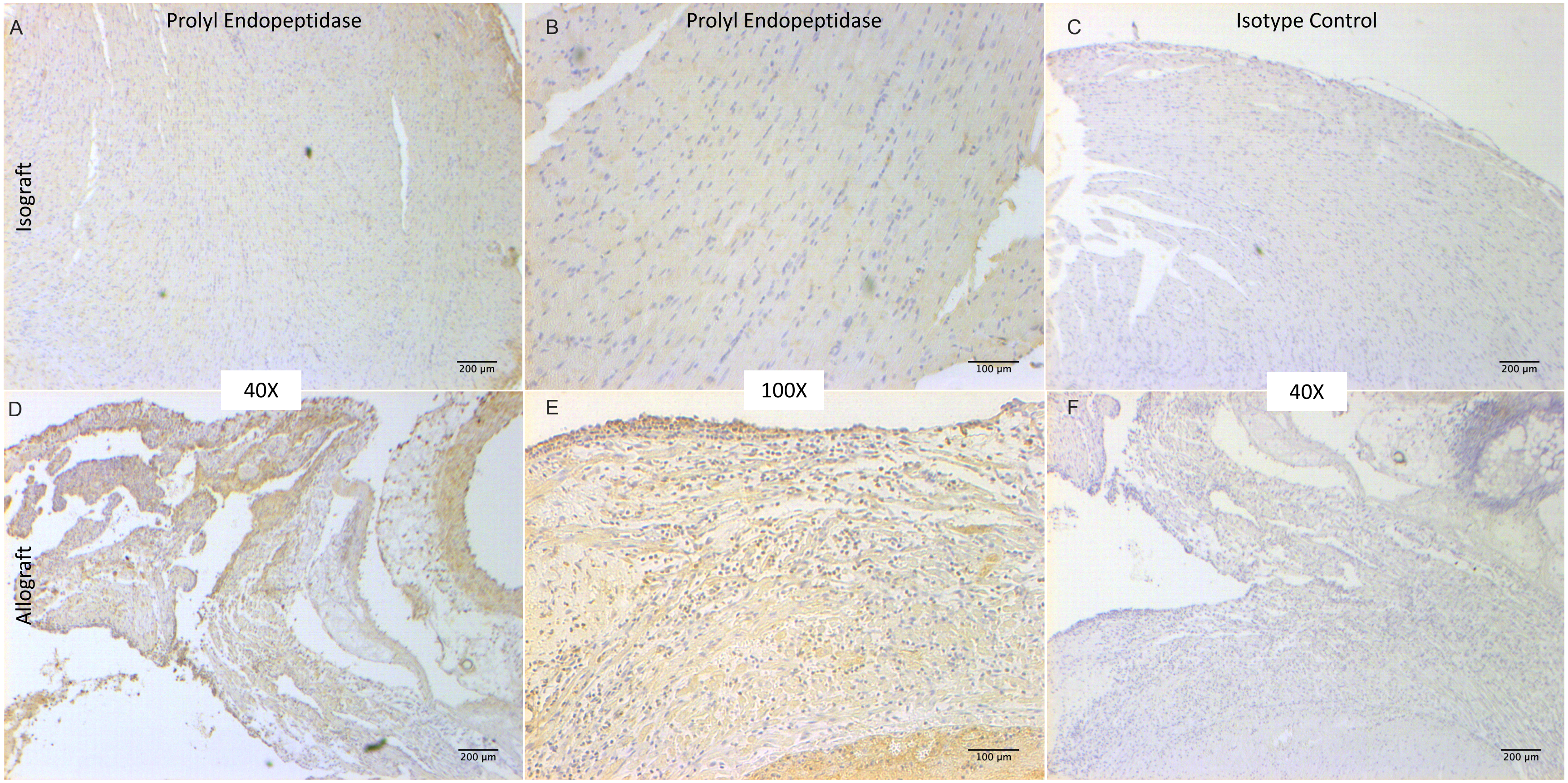

Supplemental Figure 2. Acute allograft transplant rejection induces local PE expression. 8-10 week-old, male, BALB/cj mice received heterotopic heart transplants from similarly aged BALB/cj (isograft) or C57BL/6j (allograft) donor mice. Mice were sacrificed 3 days post-transplant. **A-F** illustrates representative images of myocardial tissue sections immunohistochemically stained for PE or an isotype control. Compared to syngeneic isografts (**A and B**), allografts were observed to have significant immune cell infiltration that was associated with a marked increase in extracellular PE expression (**D and E**).

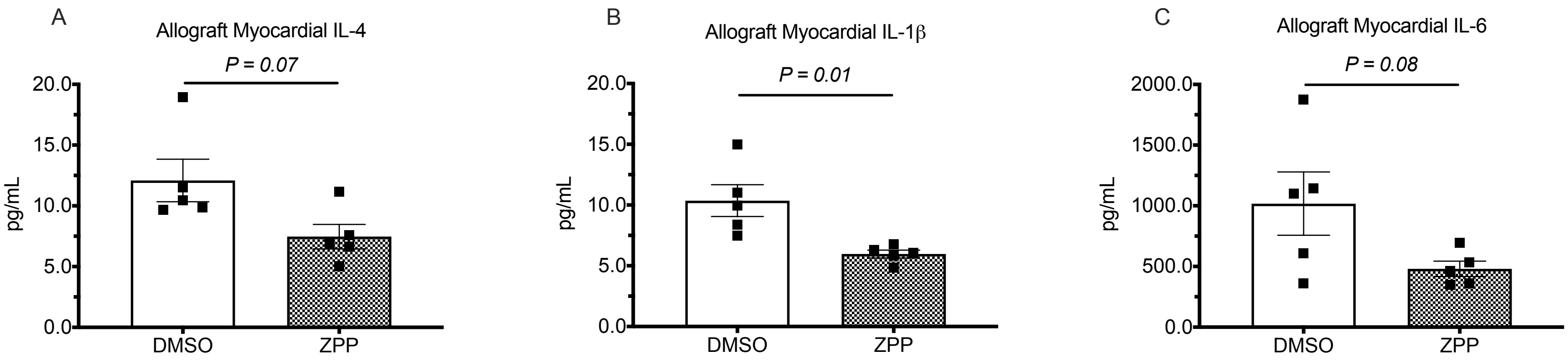

Supplemental Figure 3. PE inhibition attenuates allograft inflammatory response. Allograft mice were treated with Z-Pro-Prolinal (ZPP, 10mg/kg i.p.) or a 2% DMSO vehicle control. Compared to DMSO-treated mice, ZPP reduced key pro-inflammatory mediators (**Figure 2**). The Th2 promoting cytokine IL-4 showed a non-significant reduction with ZPP (**A**). Interestingly, the innate cytokine IL-1β was also significantly reduced (**B**) and associated with a trend towards reduced IL-6 production (**C**). *n* = 5 animals/group. Results presented as mean ± SE. Student's unpaired t-test was used for each comparison.
